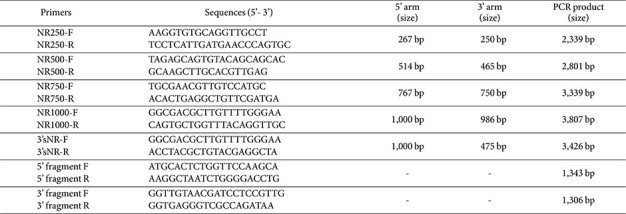# Quantitative Evaluation of NR Locus-Targeted Nuclear Transformation in *Chlorella vulgaris* Using eGFP and Flow Cytometry

**DOI:** 10.4014/jmb.2605.05007

**Published:** 2026-07-15

**Authors:** Min-Kwan Sung, So-Hyeon Jo, Tae-Jin Choi

**Affiliations:** Department of Microbiology, Pukyong National University, Busan, Republic of Korea

**Keywords:** Antibiotic-free selection, *Chlorella vulgaris*, Homologous recombination, Nitrate reductase locus, Nuclear transformation

## Abstract

*Chlorella vulgaris* is a haploid green microalga with cost-effective cultivation, high biomass productivity, and heterotrophic growth capability, making it an attractive platform for food, pharmaceutical, and recombinant protein production. However, development of efficient nuclear transformation systems has been limited by its rigid cell wall and intrinsically low homologous recombination (HR) activity. Here, we established an antibiotic-free nuclear transformation strategy targeting the endogenous nitrate reductase (NR) locus in *C. vulgaris* PKVL7422 using donor constructs designed to promote homologous recombination. Transformation outcomes were quantitatively evaluated by flow cytometry based on GFP-positive frequency. A codon-optimized GFP expression cassette driven by the heterologous CaMV 35S promoter and terminated by the *RBCS2* 3′ untranslated region was flanked by *NR* homology arms and introduced as linear donor DNA via electroporation. GFP-positive frequency was quantified by flow cytometry. GFP-positive cells were detected at frequencies of 5.1–6.3%. Optimization of homology arm length, donor DNA amount, and electroporation voltage enabled reproducible enrichment of targeted GFP integration events. Junction PCR confirmed precise insertion at the *NR* locus. Compared with previous reports, the GFP-positive frequencies observed in this study were substantially higher under our experimental conditions. Disruption of the endogenous *NR* gene enabled antibiotic-free selection based on nitrate metabolism, while GFP fluorescence allowed rapid quantitative screening. These results provide a basis for HR-mediated nuclear genome engineering and recombinant protein expression in *C. vulgaris*.

## Introduction

*Chlorella vulgaris* is a unicellular haploid microalga recognized for its rapid growth, scalability, and cost-efficient cultivation, making it an attractive platform for recombinant protein production and industrial biotechnology [1]. Its potential has been further validated by recent studies demonstrating high-level expression of viral antigens, including viral hemorrhagic septicemia virus (VHSV) glycoprotein and white spot syndrome virus (WSSV) VP28, establishing *C. vulgaris* as a feasible oral vaccine delivery system [2, 3]. These advances position *C. vulgaris* as a promising algal biofactory; however, they also expose a critical limitation: transgene expression remains highly dependent on random genomic integration, resulting in unpredictable expression stability and yield.

Despite ongoing methodological improvements, genetic transformation of *C. vulgaris* remains technically constrained. The rigid cell wall limits efficient DNA uptake, requiring optimized delivery approaches such as electroporation [4]. Although electroporation is widely used due to its simplicity and adaptability [1, 4], and transformation efficiency can be enhanced through vector modifications such as insert fragmentation [5], these strategies predominantly lead to non-specific integration events. Random insertion into the nuclear genome commonly occurs via non-homologous end joining (NHEJ), producing positional effects, gene silencing, and variable transgene expression levels [6].

Compared with bacterial expression systems such as *Escherichia coli*, nuclear expression in microalgae enables post-translational modifications (PTMs), making it attractive for the production of complex recombinant proteins. For applications requiring stable and reproducible protein production, site-specific integration is essential because it improves transgene stability and expression consistency. Homologous recombination (HR) offers a controlled alternative to random insertion; however, HR efficiency in the nuclear genome of microalgae remains intrinsically low compared with chloroplast systems [7]. Although genome-editing technologies have expanded engineering possibilities, consistent and quantitative evaluation of HR-mediated nuclear integration in *C. vulgaris* remains limited. Moreover, systematic assessment of key determinants such as homology arm length, donor DNA concentration, and electroporation voltage has not been comprehensively performed in a marker-free context.

Addressing this gap is crucial for transitioning *C. vulgaris* from a proof-of-concept expression host to a predictable and engineering-ready platform. In the present study, we designed a donor construct in which the green fluorescent protein (GFP) gene was flanked by the 5′ and 3′homologous regions of the endogenous nitrate reductase (NR) locus to promote targeted nuclear integration. While the NR locus has been previously utilized for recombinant expression in *Chlorella* [2, 3, 5], a quantitative and comparative evaluation of GFP-positive frequency under systematically varied transformation parameters has not been reported. Here, we provide a marker-free, flow cytometry-based assessment of NR locus-directed GFP-positive frequency across different homology arm lengths, donor DNA amounts, and electroporation voltages. This work establishes an experimental framework for rational optimization of targeted nuclear transformation in *C. vulgaris* and advances the development of reliable, site-specific genetic engineering strategies for microalgal biotechnology.

## Materials and Methods

### Algal Strain and Culture Conditions

*Chlorella vulgaris* strain PKVL7422, isolated in our laboratory, was cultivated in BG-11 medium supplemented with 0.5% (w/v) glucose under continuous illumination (50 μmol m^−2^ s^−1^). After 3-5 days, 1 mL of cell suspension was inoculated into 25 mL fresh BG-11 and grown to a density of approximately 1 × 10^7^ cells mL^−1^ for transformation. Transformants were maintained in BG-11-based BGNK medium supplemented with 200 mM KClO_3_ and NH_4_Cl for selection as described earlier [5]. NH_4_Cl was included only during the initial 3 days after electroporation to reduce the metabolic burden associated with nitrate assimilation and to support cellular recovery.

### Vector Construction

**Fig. 1:**
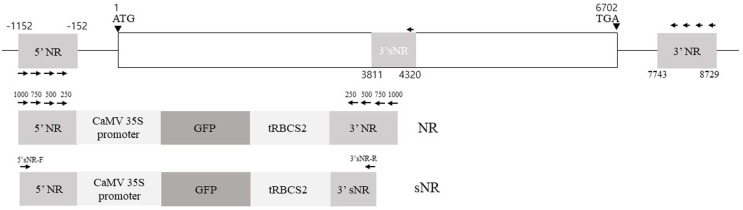
Schematic representation of the genomic organization of flanking arms and insert constructs used for *Chlorella vulgaris* transformation. Diagram illustrating the arrangement of the 5′ and 3′ flanking arms and the insert cassette. Upstream and downstream nucleotide positions are indicated relative to the adenine of the ATG start codon, which is designated as +1. Arrows denote the locations of primers used to amplify the insert DNA from the vector backbone. NR and sNR indicate constructs containing different 3′ flanking arms. The diagram is not drawn to scale.

The procedures for the construction of the plasmid pCCGFP harboring the eGFP gene is shown in Fig. S1. The pCCWVP28 vector [2, 8] was used as the backbone for nuclear genome targeting in *C. vulgaris* PKVL7422. The VP28 gene was removed by XhoI and BamHI digestion and replaced with a codon-optimized GFP gene. The GFP coding sequence was designed and codon-optimized using GeneCrafter™ software (Bioneer, Republic of Korea) according to the codon usage table of *C. vulgaris* PKVL7422. The resulting GFP expression cassette, driven by the CaMV 35S promoter and terminated by the *RBCS2* 3’ untranslated region, was amplified using the primer sets listed in Table 1 and shown in Fig. 1 and used for insertion into the NR locus by double homologous recombination.

### Optimization of Transformation

The effect of flanking arm length on GFP-positive frequency was investigated by generating insert DNAs of different sizes. These inserts were amplified by PCR using various primer sets located within the 5′ NR and 3′ NR arms, as shown in Fig. 1. The length of each arm ranged from 0.25 to 1 kb. Detailed information on the 5′ and 3′ primers and the corresponding PCR products is provided in Table 1. PCR was performed under the following conditions: an initial denaturation at 95°C for 5 min; 35 cycles of denaturation at 95°C for 20 sec, annealing at 60°C for 20 sec, and extension at 72°C for 2 min and 30 sec; followed by a final extension at 72°C for 10 min. To determine the optimal amount of DNA required for efficient transformation, 1, 5, 10, 15, or 20 μg of PCR product was used to transform 1 × 10^7^ cells in a total volume of 400 μL. Transformation was performed at 0.5, 1.0, 1.5, or 2.0 kV to identify the optimal voltage conditions. In addition, an alternative 3′ homologous arm, designated 3′sNR (Fig. 1), was designed to evaluate the effect of the distance between the two recombination sites on GFP-positive frequency after KClO_3_ selection. This configuration allowed adjustment of the spacing between the 5′ and 3′ homologous arms without altering the insert size or homology arm length.

### Transformation by Electroporation

Exponentially growing cells (1 × 10^7^) were harvested by centrifugation at 2,600 × g for 10 min, resuspended in 1 mL of osmotic buffer (0.2 M Dmannitol, 0.2 M D-sorbitol), and incubated on ice for 1 h. The cells were subsequently collected by centrifugation at 4,000 × g for 10 min and gently resuspended in 400 μL of electroporation buffer (0.5 M NaCl, 0.2 M D-mannitol, 0.2 M D-sorbitol, 20 mM MOPS, 5 mM CaCl_2_, and 5 mM KCl; pH 7.2). Insert DNAs were generated by PCR using the primer sets listed in Table 1, quantified using a NanoDrop™ One/OneC spectrophotometer (Thermo Fisher Scientific, USA), and purified prior to use. Linear DNA (1−20 μg) was added to the cell suspension, and the mixture was incubated on ice for 10 min before being transferred to a 0.2-cm gap electroporation cuvette. Electroporation was carried out using a Gene Pulser II system (Bio-Rad, USA) at 0.5−2.0 kV, 25 μF, and 400 Ω. Immediately after pulsing, the cells were transferred to 2 mL of BGNK medium and incubated in the dark at 20°C for 18 h. The cultures were then grown in 5 mL of BGNK medium under light conditions for 3 days prior to analysis.

### Flow Cytometry Analysis

The GFP-positive frequency after KClO_3_ selection was determined by quantifying GFP-positive cells using flow cytometry. Wild-type and transformant cells were adjusted to a concentration of 5 × 10^6^ cells mL^−1^ in 1 × PBS (final volume, 1 mL), washed three times by centrifugation (4,000 × g, 5 min, 4°C), and resuspended in PBS prior to analysis. Samples were analyzed using a BD FACSVerse™ flow cytometer (BD Biosciences, USA) equipped with a 488 nm excitation laser and a 527/32 nm emission filter (FITC channel). Cellular debris was excluded by gating on SSC-A versus FSC-A, and doublets were discriminated using FITC-W versus FITC-A. The fluorescence threshold was established based on wild-type cells, excluding >99% of the negative population. GFP-positive cells were quantified per 10,000 recorded events.

### Confirmation of Site-Specific Integration

Five colonies were randomly selected from transformants grown on BGNK agar plates, and genomic DNA was extracted from wild-type and selected transformants from optimized transformation conditions (1 kb arms, 1 μg DNA and 1 kV) using a Plant DNA Extraction Kit (SCINOMICS, Republic of Korea). Junction PCR was performed to confirm homologous recombination at the NR locus using primer pairs spanning the 5′ and 3′ integration junctions with corresponding primer sets listed on Table 1. PCR was conducted with LF Master Mix (Elpis Biotech, Republic of Korea) with an initial denaturation at 95°C for 5 min, followed by 35 cycles of denaturation at 95°C for 10 sec, annealing at 59°C for 20 sec, and extension at 72°C for 1 min, with a final extension at 72°C for 5 min, and amplicons were analyzed by electrophoresis on 1% agarose gels.

### Statistical Analysis

Data was obtained from three independent biological replicates. Statistical analyses were performed using JASP (version 0.95.4). Differences among groups were evaluated by one-way ANOVA followed by Tukey’s post hoc test. Results are presented as mean ± standard deviation (SD), and differences were considered significant at *p* < 0.05.

## Results and Discussion

### Optimization of GFP-Positive Frequency in *C. vulgaris*

**Fig. 2:**
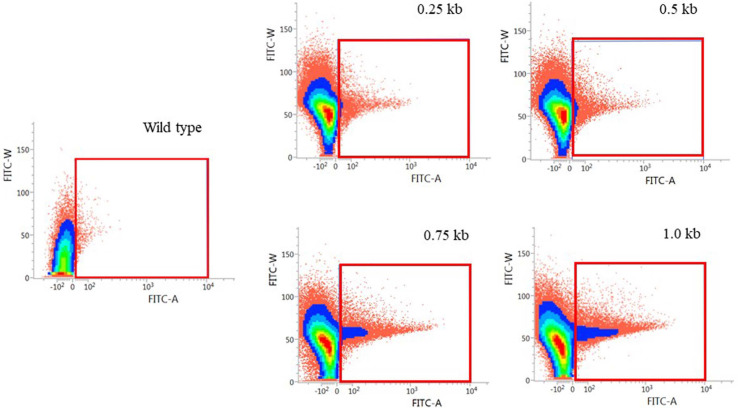
Flow cytometric analysis of *Chlorella vulgaris* cells transformed with constructs containing different flanking arm lengths. Representative flow cytometry histograms showing green fluorescence intensity in *C. vulgaris* cells transformed with DNA constructs containing 0.25 kb, 0.5 kb, 0.75 kb, or 1.0 kb flanking arms. Wild-type (WT) cells were used as a negative control to define the background fluorescence threshold. GFP-positive populations were gated by excluding 99% of the WT fluorescence signal.

**Fig. 3:**
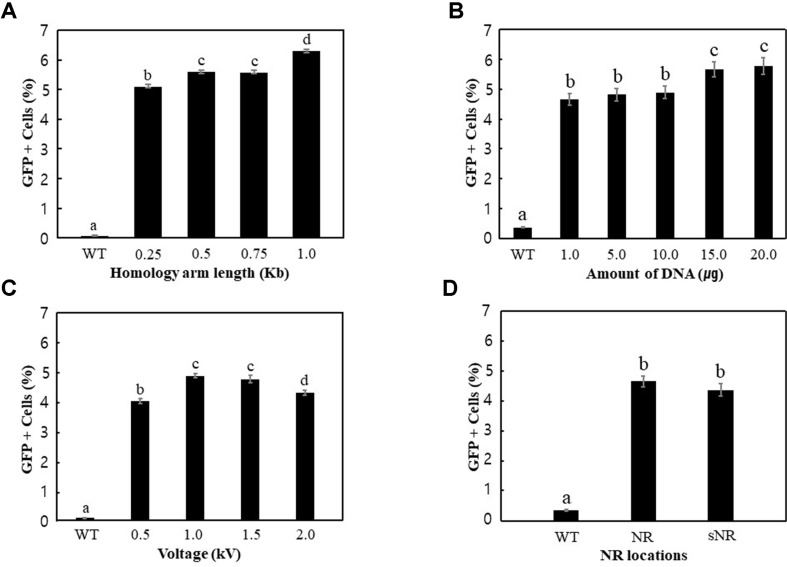
Optimization of transformation parameters in *Chlorella vulgaris*. The percentage of GFP-positive cells (excluding 99% of the wild-type fluorescence background) was measured following transformation under different conditions: varying flanking arm lengths (**A**), amounts of added DNA (**B**), electroporation voltages (**C**), and 3′ arm integration sites (**D**). Different superscript letters indicate statistically significant differences (*p* < 0.05). Error bars represent the standard deviation (SD) of three independent replicates. The genomic locations of the 3′ arms are shown in Fig. 1.

To optimize electroporation-mediated transformation in *C. vulgaris*, GFP-positive frequency was systematically evaluated by varying key parameters, including homology arm length, donor DNA concentration, applied voltage, and the location of 3’ flanking arms. GFP-positive frequency was determined as the percentage of GFP-positive cells among 10,000 recorded events. A fluorescence threshold was established to exclude >99% of the wild-type population (Fig. 2). Representative flow cytometry histograms are shown in Fig. S2. GFP-positive frequency increased as the length of the homologous flanking arms was extended from 0.25 kb to 1.0 kb (Fig. 3A). Significant differences were observed between 0.25 kb and 0.5−0.75 kb, and a further significant increase was detected at 1.0 kb, which showed the highest GFP-positive frequency.

Homology arm length is a critical parameter influencing the efficiency of DNA integration via HR. A previous study in mouse embryonic stem (ES) cells [9] showed that gene-targeting frequency increased markedly when total homology length was extended from ~1.3 kb to ~6.8 kb. Homology arm length remains an important determinant of HDR efficiency in nuclease-assisted genome editing, such as CRISPR/Cas9-mediated knock-in. A previous study [10] reported that increasing homology arms up to approximately 1−2 kb enhanced targeted integration efficiency and increased the frequency of biallelic events in mouse ES cells. However, some reports suggest that extremely long homology arms do not always proportionally increase targeting efficiency. For example, studies comparing conventional (~8 kb) versus very long (>15 kb) homology arms in EScell targeting have found no significant improvement in recombination frequency [11]. Moreover, the minimal homology requirement varies substantially among organisms. In contrast to mammalian systems that longer arms are typically required due to the predominance of NHEJ, highly efficient HR allows integration with as little as 40−50 bp of flanking homology in budding yeast [12]. The results of this study demonstrate that homology arm length significantly affects HR-mediated DNA integration efficiency. These findings are consistent with the general principle that longer homology arms improve recombination efficiency by promoting more stable homologous pairing during DNA repair.

Similar observations have been reported in microalgae, including *Chalmydomonas reinhardtii*, where homology-directed genome engineering approaches rely on sufficiently long homologous sequences to facilitate efficient targeted integration [13, 14]. However, quantitative studies evaluating factors that influence HR efficiency in *C. vulgaris* remain limited. Therefore, the results of this study provide valuable insights into the optimization of HR-mediated genome engineering in this species. GFP-positive frequency increased with increasing homology arm length, reaching the highest level at 1.0 kb. Considering both recombination efficiency and cloning feasibility, homology arms in the range of 0.5–1.0 kb may represent a practical design for genome engineering applications in similar systems. In comparison, transformation efficiencies reported for the microalga *C. reinhardtii* are typically 2–6 × 10^3^ transformants per μg DNA when cells retain their cell wall, and approximately 10^5^ transformants per μg DNA when the cell wall is removed [15]. Therefore, the GFP-positive frequencies observed in the present study compare favorably with those reported previously.

### Effect of Donor DNA Amount on GFP-Positive Frequency

Electroporation-mediated transformation of *Chlorella* cells showed a dose-dependent increase in efficiency as donor DNA concentration was raised from 1 μg to 20 μg (Fig. 3B). The percentage of GFP-positive cells increased with increasing donor DNA input, showing a noticeable improvement up to 15 μg. However, only a marginal additional increase was observed at 20 μg, suggesting that GFP-positive frequency approaches a plateau at higher DNA amounts.

In microalgae such as *Chlorella*, electroporation enhances membrane permeability, allowing exogenous DNA to enter the cell; thus, GFPpositive frequency depends on both effective DNA uptake and subsequent intracellular DNA processing. Increasing donor DNA concentration raises the probability that sufficient plasmid or repair template molecules enter the cell and persist long enough to undergo genomic integration. Consistent with this, dose-dependent increases in knock-in or transformation frequency have been widely reported in microalgal systems, with efficiency improving as DNA amounts increase until a saturation threshold is reached [16, 17].

However, although higher donor DNA levels can enhance GFP-positive frequency, this effect is not unlimited. The plateau observed at elevated DNA concentrations indicates that donor availability eventually ceases to be the primary limiting factor. Instead, other constraints such as reduced cell viability following electroporation, limited nuclear import efficiency, chromatin accessibility, or the balance between homologous recombination HR and NHEJ may become rate-limiting. In many eukaryotic microorganisms, NHEJ predominates, reducing the proportion of precise integration events even when donor DNA is abundant [11]. Furthermore, excessive DNA amounts may increase cytotoxicity, impose metabolic burden, and substantially elevate preparation costs, potentially offsetting gains in overall efficiency.

Taken together, these findings indicate that while increasing donor DNA enhances electroporation-mediated transformation in *Chlorella*, the response reaches a practical optimum. Although approximately 15 μg yielded maximal efficiency under the present conditions, considering costeffectiveness and potential cytotoxicity, a donor DNA range of 1−5 μg may represent a practical and efficient balance for achieving stable transgene integration [18, 19]. Further improvements are therefore more likely to arise from optimization of electroporation parameters or targeted modulation of DNA repair pathways rather than simply increasing donor DNA concentration.

### Effect of Electroporation Voltage on GFP-Positive Frequency

Electroporation voltage significantly influenced GFP-positive frequency in *Chlorella* cells. GFP-positive frequency increased from 0.5 kV to 1.0 kV, reaching a maximum at 1.0 kV. A slight decrease was observed at 1.5 kV, and a further reduction occurred at 2.0 kV, although efficiencies remained higher than at 0.5 kV (Fig. 3C). These results indicate the presence of an optimal voltage (approximately 1.0 kV) for maximizing GFPpositive frequency under the present conditions.

Voltage optimization is a critical determinant of electroporation-mediated GFP-positive frequency in *C. vulgaris*. Electroporation facilitates DNA uptake by generating transient pores in the cell membrane, and increasing voltage enhances membrane permeabilization, thereby promoting DNA entry. Previous studies in *C. vulgaris* have reported maximal GFP-positive frequency at 0.8−1.2 kV, with a marked reduction in cell viability above 1.5 kV, a pattern consistent with the voltage-dependent response observed in the present study [20]. Cell viability was closely associated with the applied voltage: at lower voltages, the electric field strength was presumably insufficient to reach the critical threshold required for effective pore formation, resulting in reduced DNA uptake and low GFP-positive frequency. In contrast, higher voltages likely induced excessive and irreversible membrane damage, leading to substantial cellular injury and decreased survival [21].

The bell-shaped relationship between voltage and GFP-positive frequency observed here aligns with previous reports in microalgae, where efficiency increases with electric field strength up to an optimal threshold, beyond which cell survival declines [16, 17]. In eukaryotic microalgae, successful transformation depends not only on membrane permeabilization and DNA entry, but also on subsequent intracellular trafficking, nuclear import, and DNA repair processes. High-voltage pulses may impair cellular recovery mechanisms and limit homologous recombination or stable genomic integration, further reducing overall GFP-positive frequency.

Overall, these findings demonstrate that electroporation voltage is a key parameter in *Chlorella* transformation, requiring a balance between sufficient membrane permeabilization and maintenance of cell viability. Under the conditions tested, 1.0 kV provided optimal GFP-positive frequency. Further improvements may be achieved through combined optimization of pulse duration, DNA concentration, and the physiological state of the cells.

### Effect of 3′ Insertion Site Distance on GFP-Positive Frequency

To evaluate the effect of the 3′ insertion site on homologous recombination efficiency in *Chlorella*, two constructs targeting different 3′ regions were compared under identical conditions (1 kb homology arms; 4 kb donor cassette), depicted as NR and sNR. Both NR and sNR inserts produced substantially higher percentages of GFP-positive cells than the WT control. NR showed slightly higher GFP-positive frequency than sNR, although the difference was moderate (Fig. 3D).

The primary distinction between the two targets was the genomic distance between the recombination arms. In NR, the distance between the two arms (~6 kb) was approximately twice the size of the donor fragment (4 kb), whereas in sNR the distance (~2 kb) was similar to the donor size. Because homology arm length and donor cassette size were identical between the two constructs, the observed difference may reflect the influence of genomic spacing and locus-specific genomic characteristics on the recombination process.

However, the difference in GFP-positive frequency between NR and sNR was relatively small, suggesting that genomic spacing alone is unlikely to be a major determinant of transformation outcome under the conditions tested in this study. HR requires strand invasion and alignment between donor and chromosomal sequences. The physical distance between recombination junctions can affect repair outcomes, particularly when large genomic segments must be replaced or reorganized. Studies in mammalian cells have shown that recombination efficiency depends not only on homology length but also on the spatial organization of the target locus [9, 10]. In yeast, chromosomal context and local topology similarly influence integration frequency even when donor design is unchanged [12].

However, a limitation of the present study is that conclusions drawn from mammalian systems may not be directly applicable to the microalga *C. vulgaris*. Previous studies have suggested that transgene integration efficiency in microalgae can be influenced by locus-dependent factors, including genomic location, safe harbor sites, genomic context, and epigenomic features [22]. In addition, position-dependent effects associated with local chromatin characteristics have been reported in genome engineering studies of *C. reinhardtii* [17]. However, studies systematically evaluating the influence of genomic location and locus-specific characteristics on homologous recombination efficiency in microalgae remain limited. Therefore, further comparative analyses targeting multiple genomic loci will be necessary to clarify the contribution of genomic context and chromatin organization to transformation outcomes in *C. vulgaris*.

### Confirmation of Homologous Recombination

**Fig. 4:**
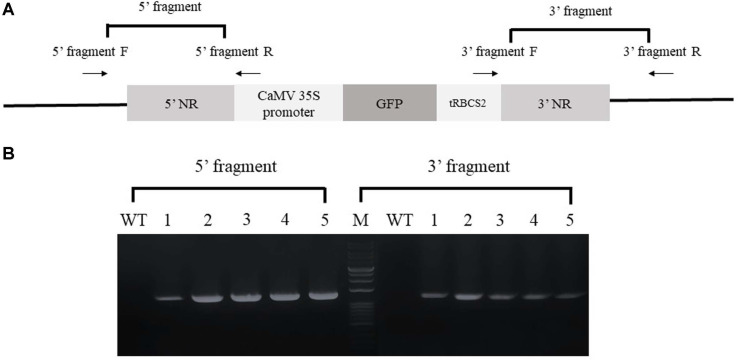
Confirmation of homologous recombination without indels in transformed *C. vulgaris*. (**A**) The expected recombination sites of the 5’ and 3’ flanking regions of wild type and randomly selected transformants were amplified with primers schematically located and listed in Table 1. (**B**) Junction PCR analysis showing that specific products were amplified only in transformants, while no amplification was detected in WT cells. WT: wild-type *C. vulgaris* PKVL7422; lanes 1–5: independent transformants; M: DNA marker (1 kb plus).

To assess whether targeted recombination resulted in unintended nucleotide insertions or deletions at the integration site, chromosomal DNA from randomly selected transformants was analyzed by junction PCR using primer sets located outside the 5′ and 3′ homology arms and spanning the recombination locus. As shown in Fig. 4, specific PCR products were detected only in transformants, whereas no amplification was observed in wild-type genomic DNA, confirming site-specific integration at the NR locus. Subsequent Sanger sequencing of these PCR products revealed no nucleotide differences at the junction regions, indicating that the recombination event occurred precisely without detectable insertion or deletion mutations at the homology arms.

Additionally, ten independent transformants were further analyzed by junction PCR and Sanger sequencing (Fig. S3). Homologous integration of the GFP expression cassette into the NR locus was confirmed in all analyzed clones, with no detectable indels at the examined recombination junctions. These results further support the conclusion that KClO_3_ selection preferentially enriched transformants carrying NR locus disruption through HR-mediated integration.

Although no nucleotide insertions or deletions were detected within the analyzed 5′ and 3′ flanking regions, unintended indel formation remains an important consideration in microalgal transformation. During DNA integration, double-strand breaks can be repaired not only by homologous recombination but also by competing pathways such as classical NHEJ and polymerase-θ-mediated end joining. These alternative repair mechanisms may introduce small insertions or deletions at junction sites, particularly when homology-directed repair is inefficient or when repair pathway balance is shifted [23, 24]. Therefore, precise junction amplification and sequencing are essential to verify the fidelity of targeted recombination events.

The use of extended homologous flanking arms is known to enhance recombination precision and reduce error-prone repair outcomes. High-efficiency homologous recombination with minimal junction mutations has been reported in several microalgae, including *Nannochloropsis* spp. [25]. Nevertheless, even when junction regions appear intact by PCR and Sanger sequencing, larger structural variations such as partial cassette rearrangements, tandem insertions, or deletions outside the amplified region may remain undetected without more comprehensive genomic analyses [26, 27].

In the present study, junction PCR and Sanger sequencing of multiple independent transformants revealed no detectable insertions or deletions within the analyzed junction regions. These results support accurate HR-mediated integration at the NR locus. The presence of a single copy NR gene in the draft genome sequence of *C. vulgaris* PKVL7422 might exclude additional copy of integrated DNA but the analysis of the entire chromosome of GFP-positive clones could clarify additional random insertions, copy-number variation, tandem integrations or structural rearrangements outside the analyzed regions cannot be completely excluded [27-29].

## Conclusion

This study optimized key parameters for electroporation-mediated transformation in *Chlorella vulgaris*. GFP-positive frequency increased with longer homology arms, higher donor DNA concentrations, and appropriate electroporation voltage, with optimal conditions observed at approximately 1.0 kb homology arms, ~1.0 kV voltage, and moderate donor DNA levels. In addition, the genomic distance between recombination arms slightly influenced recombination efficiency, indicating that locus architecture can affect integration outcomes. Molecular analyses confirmed precise targeted integration at the analyzed junctions without detectable indels at the examined junction regions. Overall, these results provide practical guidelines for improving genome engineering efficiency in *C. vulgaris* and support its application in microalgal biotechnology.

## Supplemental Materials

Supplementary data for this paper are available on-line only at http://jmb.or.kr.



## Figures and Tables

**Table 1:** Primers used for the amplification of insert DNA and the information of resulting PCR products.