# Supplementary figures and images for: Quantitative Evaluation of NR Locus-Targeted Nuclear Transformation in Chlorella vulgaris Using eGFP and Flow Cytometry
Source: J Microbiol Biotechnol. 2026 Jul 15;36:e2605007. doi: 10.4014/jmb.2605.05007 (PMC13430251; doi:10.4014/jmb.2605.05007)

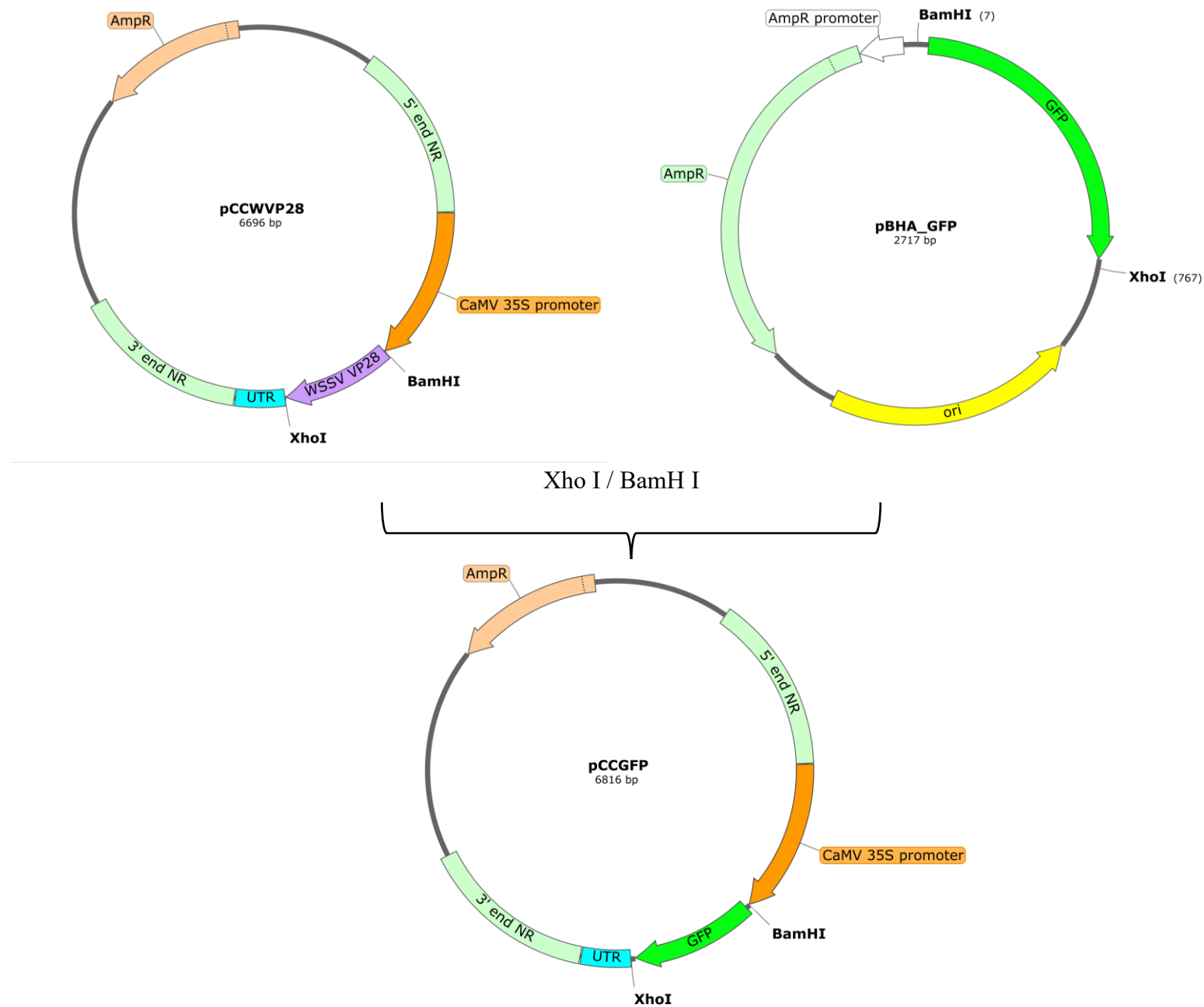

**Fig. S1**

A

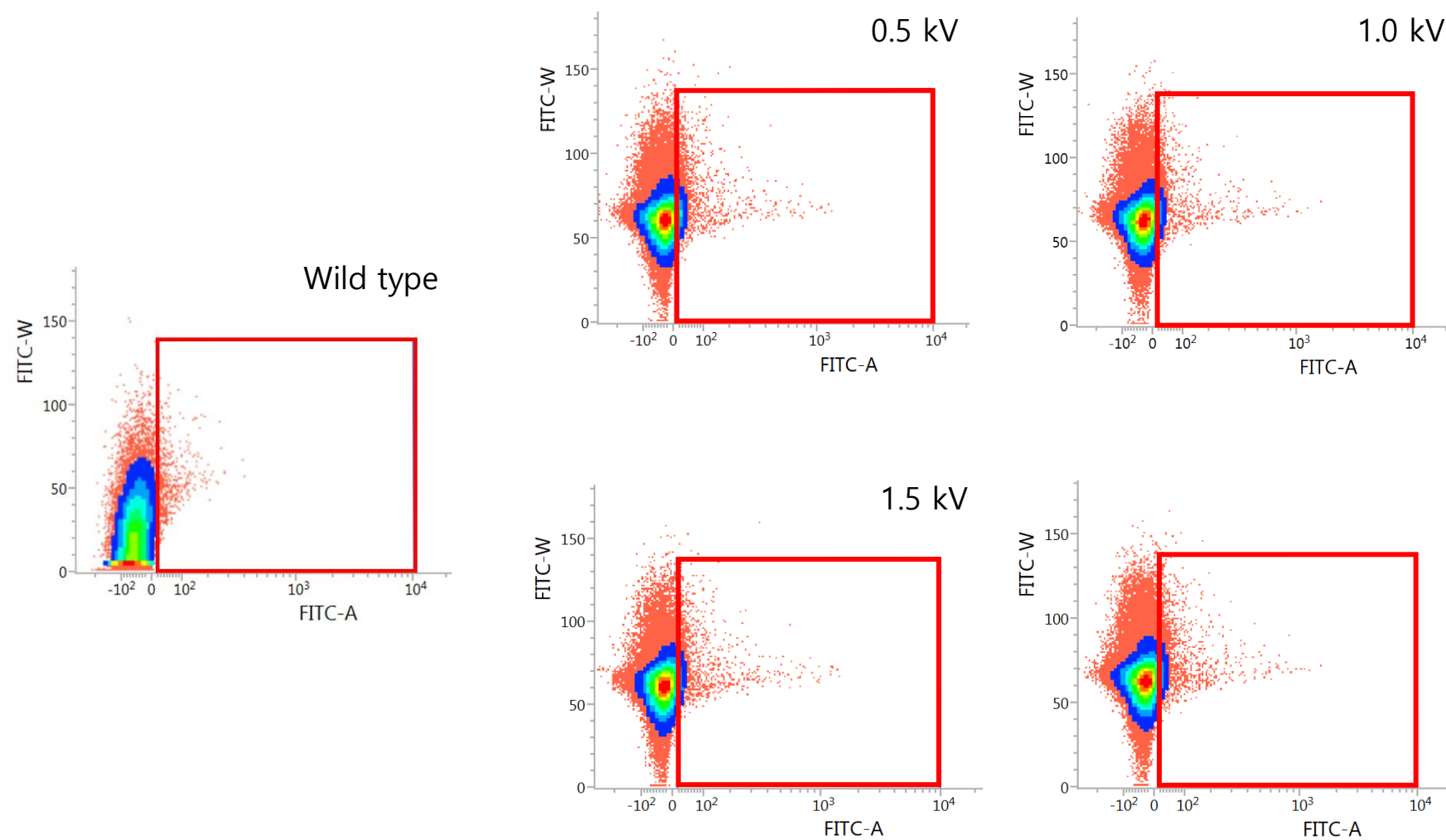

Fig. S2

B

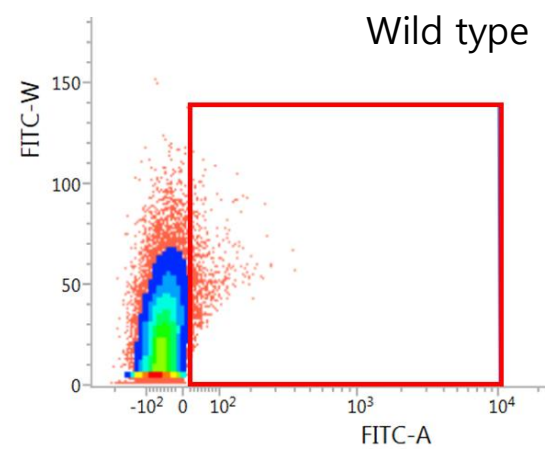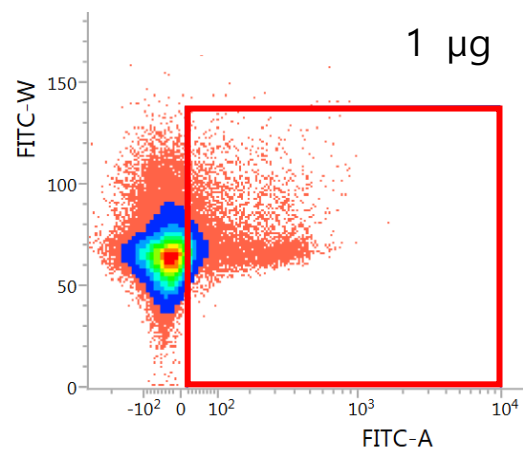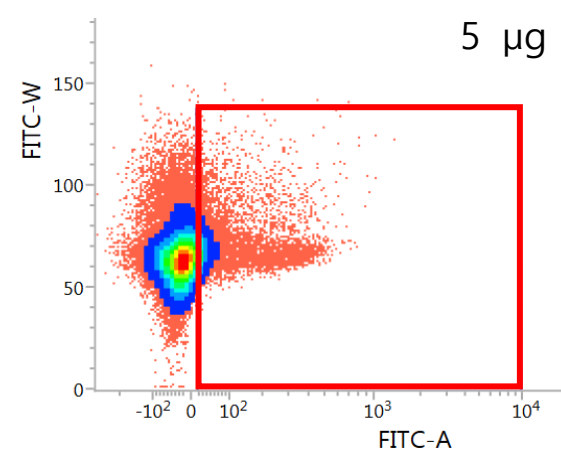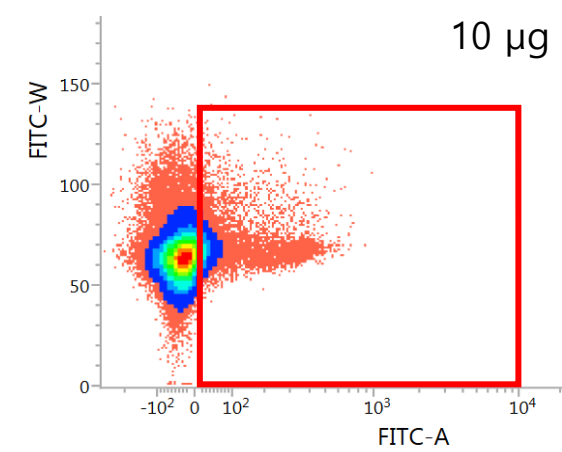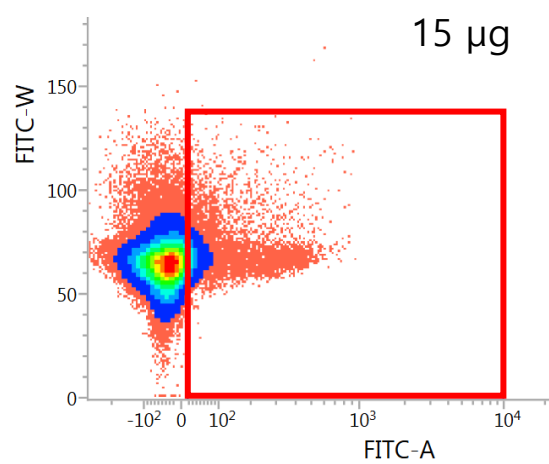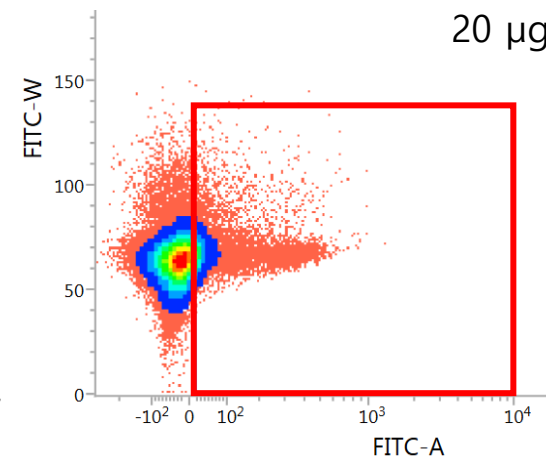

C

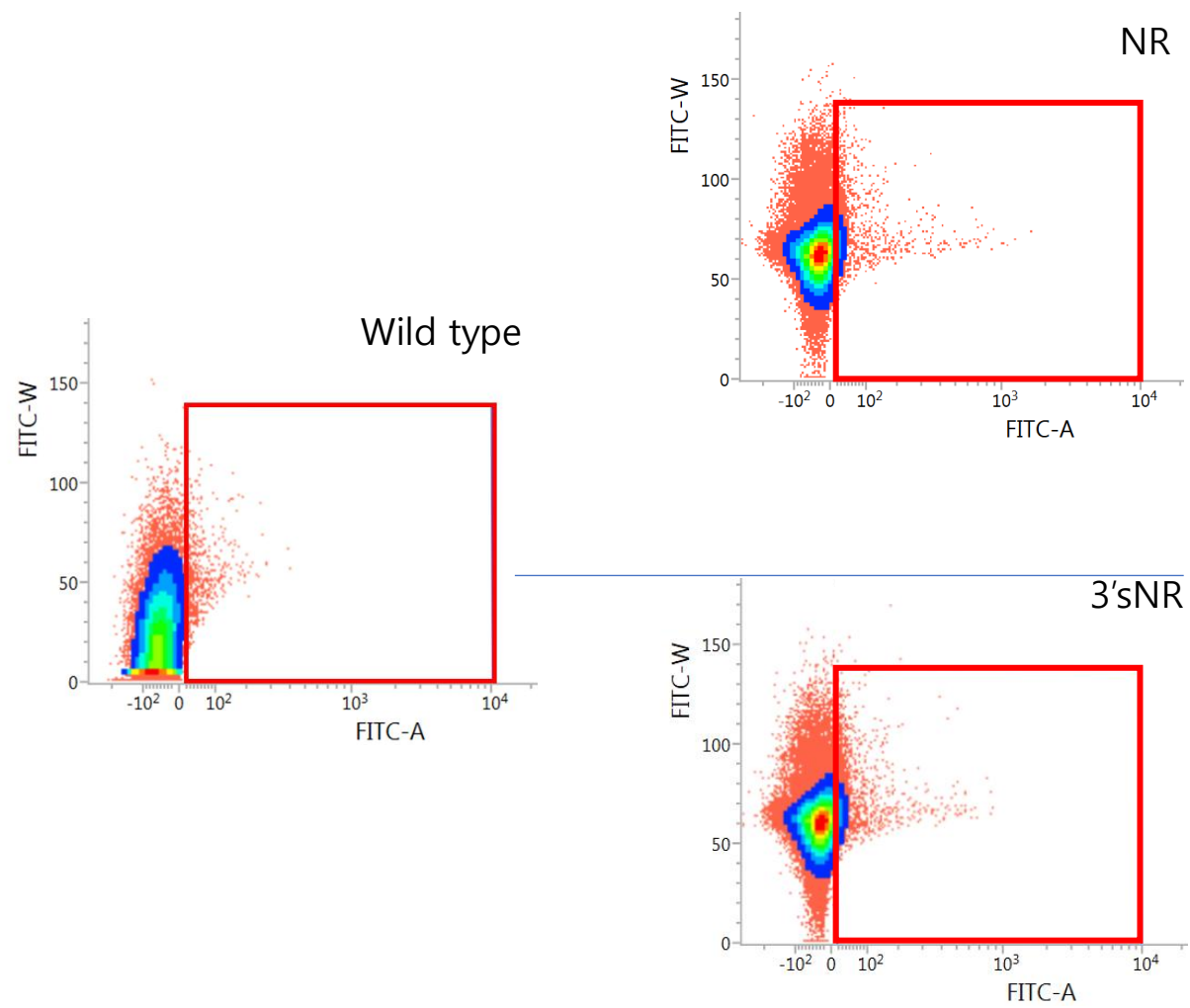

A

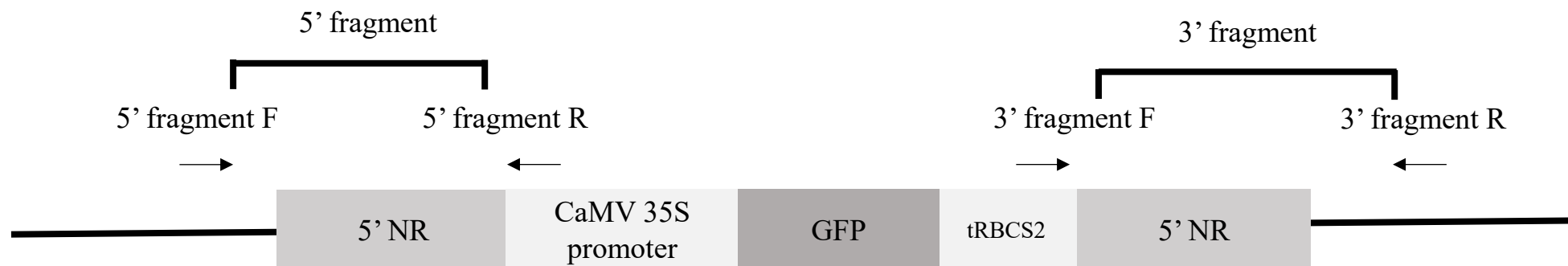

B

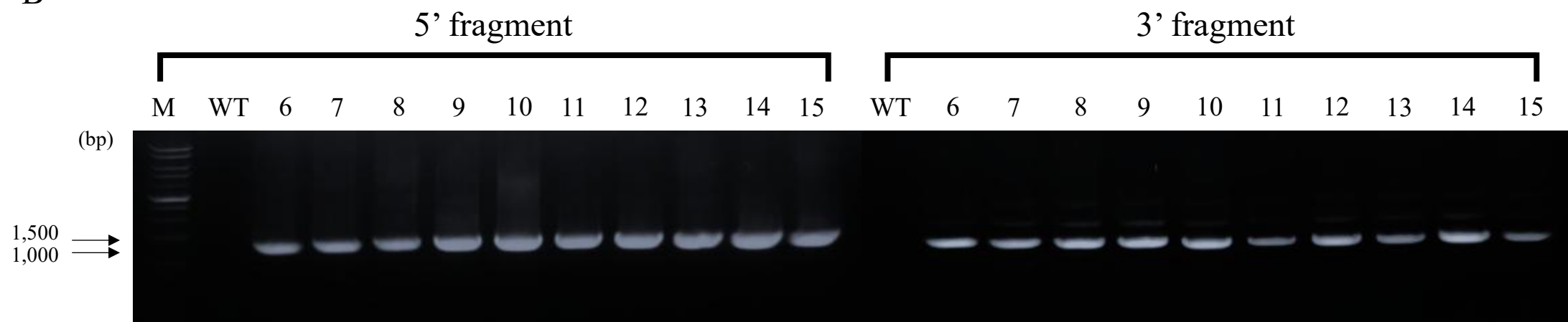

Supplement: Supplementary file 1 [file jmb-36-e2605007-supple.pdf]
